# A global perspective: Trends and insights in premenstrual disorder comorbidity research by bibliometric analysis (1999–2023)

**DOI:** 10.1097/MD.0000000000045001

**Published:** 2025-10-03

**Authors:** Xunshu Cheng, Huihao Li, Mingzhou Gao, Xiaoying Liu, Peijuan Wu, Xingping Ni

**Affiliations:** a Sichuan University of Arts and Science, Dazhou, Sichuan Province, China; b Guangzhou Xinhua University, Guangzhou, Guangdong Province, China; c Innovation Research Institute of Chinese Medicine and Pharmacy, Shandong University of Traditional Chinese Medicine, Jinan, Shandong Province, China; d Dazhou Central Hospital, Dazhou, Sichuan Province, China.

**Keywords:** bibliometric analysis, bipolar disorder, citespace, comorbidity

## Abstract

**Background:**

Premenstrual disorders represent a constellation of incapacitating gynecological disorders with numerous coexisting conditions. The identification and comprehension of disease-related comorbidities are of paramount importance in the medical field. This study aims to systematically review existing research and identify potential focus areas through bibliometric analysis.

**Methods:**

One hundred forty-nine publications on comorbidity in psychotic mood disorders between 1999 and 2023 were retrieved from the Web of Science Core Collection. The data was analyzed using bibliometric tools such as CiteSpace 6.1.R2 and VOSviewer. Specifically, the study examined the annual publication count, contributions by country and institution and details on journals, authors, citation counts, and keywords.

**Results:**

The data were retrieved on August 3, 2023. The annual number of publications showed an upward trend from 1999 to 2023. Globally, the US and Canada presented the highest publication counts and served as core research regions. Meanwhile, McMaster University and Harward University exhibited the highest output and influence by institution. In terms of author, Frey BN (McMaster University, Canada) was the most prolific with leading academic influence, while Lieb R (University of Basel, Switzerland) and Wittchen HU (Technical University Dresden, Germany) were the most cited authors. The American Journal of Psychiatry (impact factor = 17.7, 2023) was the most frequently cited journal. Furthermore, significant overlapping between premenstrual syndrome and premenstrual dysphoric disorder warrants further investigation, and the intrinsic connection between premenstrual dysphoric disorder and bipolar disorder is a rising focus. Temporally, research shifted from prevalence surveys to diagnostic and mechanistic studies.

**Conclusions:**

This bibliometric study comprehensively analyzes the current state of research on physical and mental health comorbidities. North America became a prominent leader in contributions from countries, institutions, authors, and journals. Additionally, the study underscores the potential for further exploration of comorbidity between physiological and psychiatric conditions, suggesting a promising avenue for future research efforts.

## 1. Introduction

Premenstrual disorders (PMDs) serve as a group of severe mood and behavioral disorders affecting women’s health worldwide. Mainly, premenstrual syndrome (PMS) and premenstrual dysphoric disorder (PMDD) are the primary manifestations, complicated with numerous physiological, mental, and behavioral distress related to the menstrual cycle.^[1,2]^ Among PMDs, PMDD, as a severe form of PMS, was initially categorized as “depressive disorders not otherwise specified” in Diagnostic and Statistical Manual of Mental Disorders (DSM)-IV and has since been reclassified as a distinct diagnostic entity under the “Depressive Disorders” category in the Diagnostic and Statistical Manual of Mental Disorders, Fifth Edition (DSM-5).^[3,4]^ It was also included as a part of gynecological diagnosis in the World Health Organization’s International Classification of Diseases, 11th Revision since 2019.^[5]^ Epidemiological surveys show global variations in PMD prevalence: PMS affects 12% to 98% of women worldwide,^[6,7]^ while PMDD ranges from 3% to 8% globally, with regional peaks up to 48.0%.^[8]^ Also, Many PMD cases report suicidal ideation in the late luteal phase,^[9,10]^ implying a significant socioeconomic burden.

Various psychiatric and somatic comorbidities were previously reported in PMD patients.^[11,12]^ A study on Han Chinese women with bipolar disorder (BD) showed that incidence rates of PMS and PMDD in BD are 57.6% and 20.6%, respectively. Younger patients in this cohort tended to exhibit higher scores on the Hamilton Rating Scale for Depression (17-item version) including its anxiety subscales, somatization, cognitive deficits, psychomotor delays, increased appetite, and lead-induced paralysis.^[13]^ Moreover, a genetic association study indicated that PMDs symptoms were associated with the polygenic risk scores for major depression, bipolar disorder, attention-deficit/hyperactivity disorder, schizophrenia, and autism spectrum disorder, representing an overlapping genetic cause between PMDs and psychiatric disorders.^[14]^ Comorbidities for PMDD covered a range of diseases, including female sexual dysfunction,^[15]^ eating disorder,^[16]^ irritable bowel syndrome,^[17]^ and diabetes mellitus.^[18]^

Despite growing reports of PMD comorbidities, existing research primarily focuses on single comorbidity pairs and lacks a global overview of research trends, core contributors, or evolving priorities. Bibliometrics serves as a quantitative analysis to examine scientific literature, focusing on evaluating publication and citation data, with the primary goal of gaining a deeper understanding of the structure, development, and dynamics of scientific disciplines.^[19]^ Bibliometrics addresses this gap by quantitatively analyzing publication/citation data to: map the structure of the field (e.g., core countries, institutions, and journals); track temporal evolution; identify knowledge gaps; and clarify conceptual relationships between themes. Citation metrics (e.g., citation counts, h-index, journal impact factor) further help prioritize high-impact, reproducible research—critical for guiding clinical practice and future funding.

## 2. Methods

### 2.1. Data source and retrieval strategy

**Figure 1.**
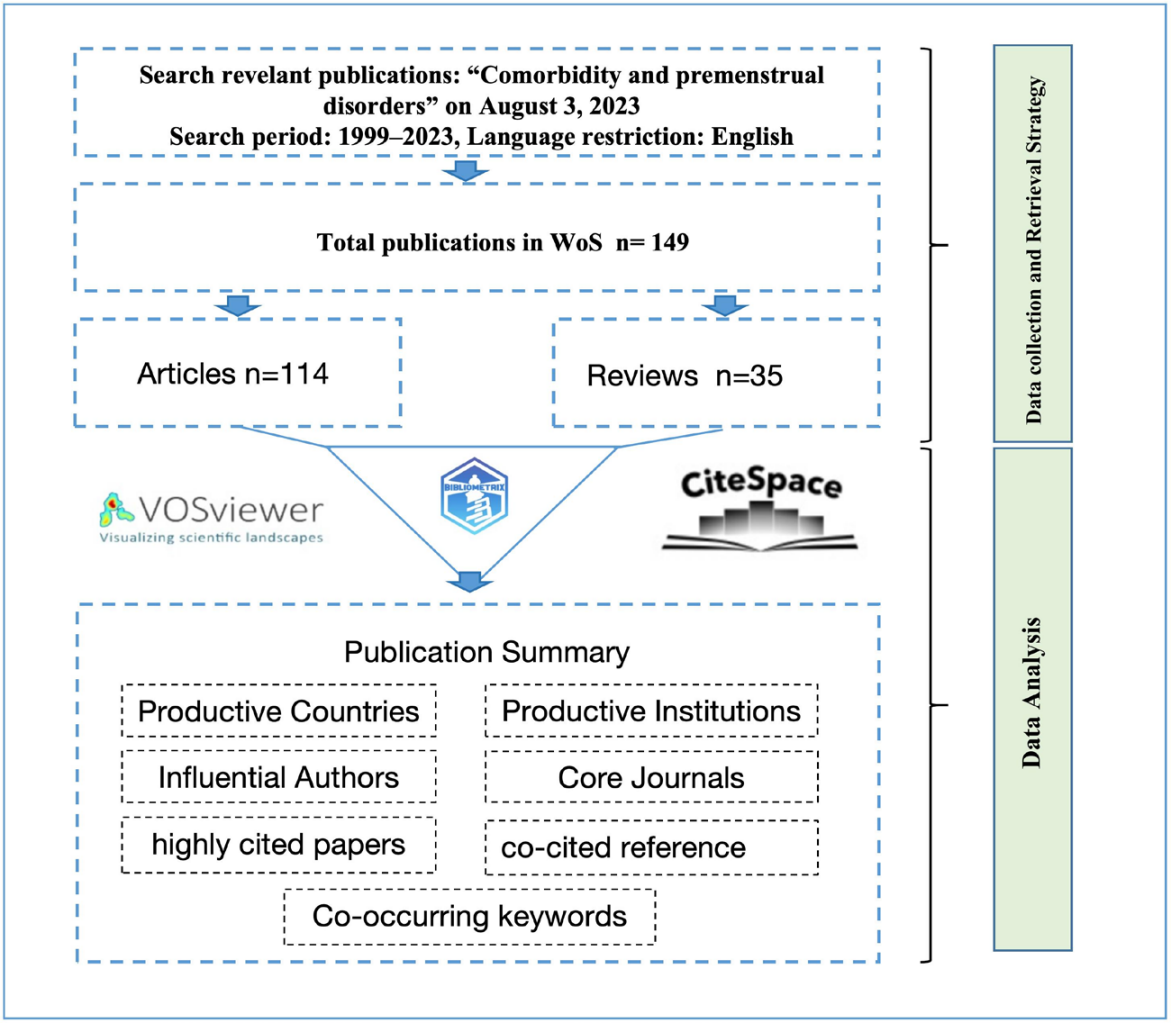
Flowchart of the screening process.

A systematic literature search was conducted from the Web of Science Core Collection (WOSCC) covering the period 1999 to 2023. A total of 149 literatures were retrieved on August 3, 2023 (single search day to avoid bias from database updates). The following specific retrieval formula for the advanced search was used: (TS = (“Premenstrual Syndrome” OR “Premenstrual Dysphoric disorder” OR “late luteal phase dysphoric disorder” OR “Premenstrual disorder”) AND (TS = multimorbid* OR comorbid* OR polymorbid* OR multimorbid* OR comorbid* OR polymorbid*). The document types were restricted to articles and review articles, and the language was limited to English. By combining it with manual screening, irrelevant documents were excluded. The scientometric research process is shown in Figure 1.

### 2.2. Data analysis and visualization

All eligible documents retrieved in WOSCC were exported in plain text format, then imported into the R package “Bibliometric” (Boston), CiteSpace 6.1.R2 software (Philadelphia), and VOSviewer (Leiden, Netherlands) for bibliometric analysis. The descriptive information on the country, institution, and author of the research topic was generated by WOOSC, and the graphics were developed based on the software’s output. Parameters in CiteSpace were set as follows: time range = 1999–2023, yearly slicing interval = 1, selection criteria = top 30 cited references per year for co-citation and keywords analysis. Node size represents the frequency of an entity. Cluster color distinguishes thematic groups. Node distance indicates the strength of association. Besides, the h-index, m-index, and g-index are metrics utilized to assess researchers’ productivity and impact in academia. The h-index signifies the number of articles a researcher cites at least h times. The g-index is a modification of the h-index, reflecting the average number of citations per article. While the h-index provides a cumulative assessment of a researcher’s output, it may disadvantage those with shorter academic careers. The m-index, the other hand, calculates the annual h-index of researchers since their initial publication.

## 3. Results

### 3.1. Annual publication outputs trend

**Figure 2.**
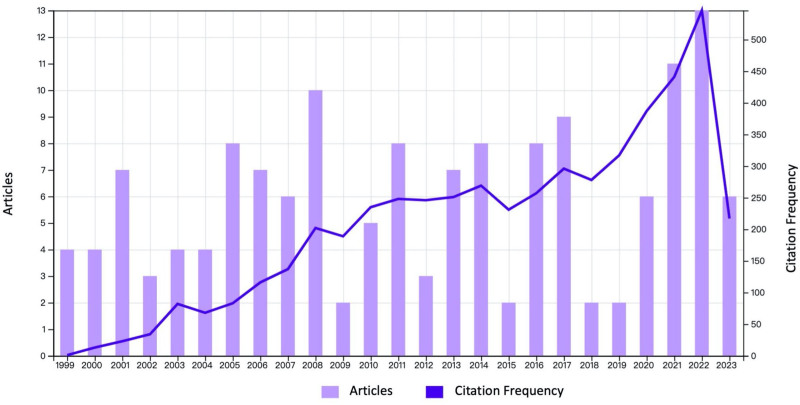
Number and citations of annual publications on PMDs and comorbidity research. PMDs = premenstrual disorders.

A total of 149 publications were retrieved from the WOSCC database. Each publication in this field has an average of 34.68 citations, and 4162 related references were involved. Figure 2 illustrates the number of annual publications and citation frequency on PMDs and comorbidity research. Since 1999, the output of PMDs and comorbidity research and the frequency of citations have gradually increased. According to the WOSCC database, an early representative publication could be traced back to “Depression in Women: Diagnostic and Treatment Considerations” Field.^[20]^ Meanwhile, the latest publication was “Premenstrual Dysphoric Disorder and sexual function: A Narrative Review,”^[15]^ the author has proposed that PMDD and female sexual dysfunction were the 2 prevalent illnesses in women.

### 3.2. Analysis of productive country or region

**Figure 3.**
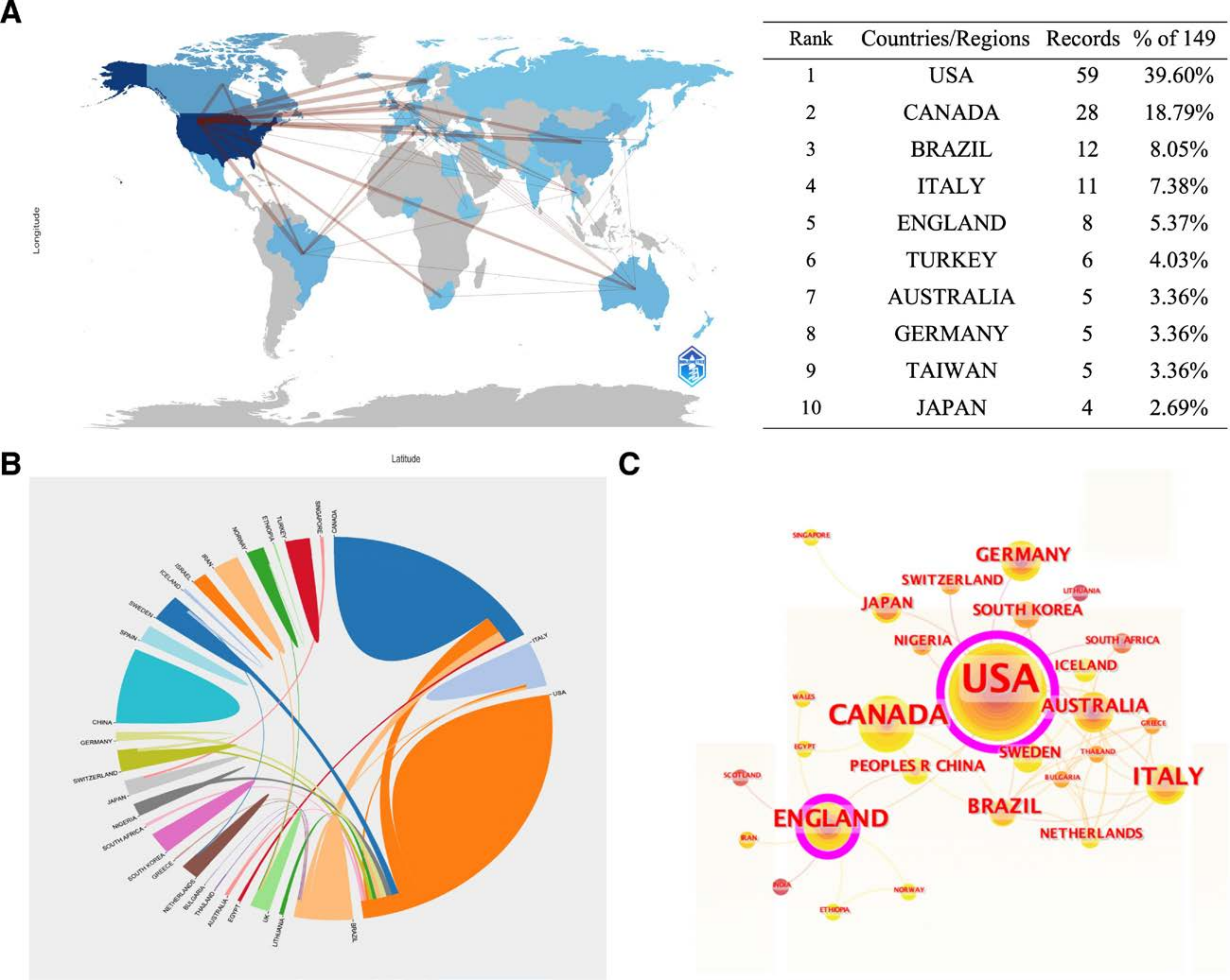
Analysis of country or regions on PMDs and comorbidity research. PMDs = premenstrual disorders.

A total of 38 countries were included in the PMDs and comorbidities research. Fifty-nine publications originated from the US, followed by Canada (28 publications), Brazil (12 publications), Italy (11 publications), England (8 publications), Turkey (6 publications), Australia (5 publications), Germany (5 publications), China Taiwan (5 publications), and Sweden (4 publications) among the top 10 productive countries (Fig. 3A). In terms of international collaboration, the US (centrality 0.82) and England (centrality 0.42) served as the 2 major research cores, with the US taking the leading position (Fig. 3C).

Interestingly, premenstrual tension was first proposed in 1931 by New York in the US, later called premenstrual syndrome.^[20]^ Since then, women with PMS or PMDD have gradually received attention, and research in this field has slowly developed. Thus, the US and Canada have steadily become high-yield countries and core regions for PMDD research, leading the research direction of the clinical condition.

### 3.3. Analysis of productive institution

**Figure 4.**
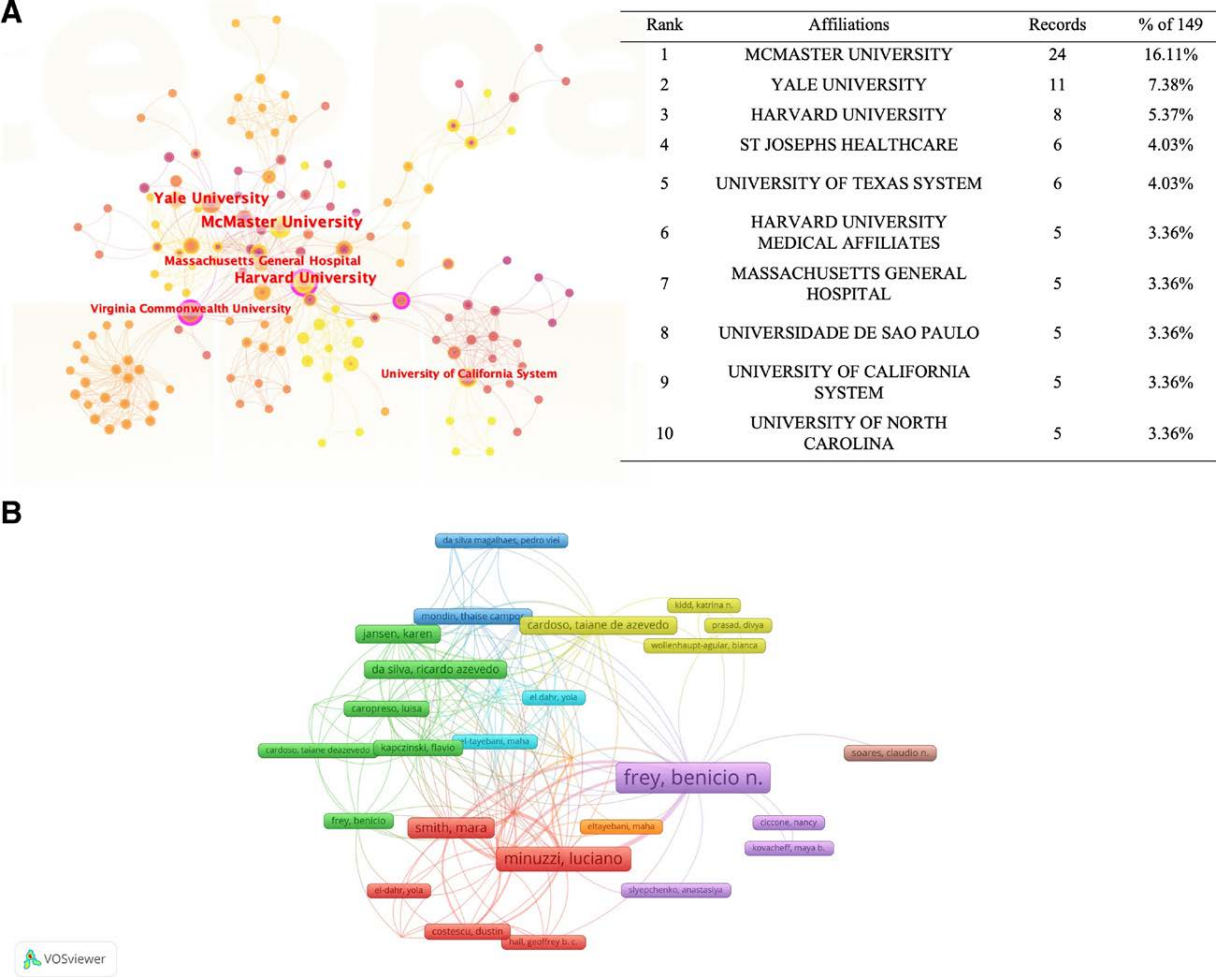
Analysis of institutions and authors on PMDs and comorbidity research. PMDs = premenstrual disorders.

Two hundred ninety-two institutions have been included in PMDs and comorbidity research. The top 10 most productive institutions are listed in Figure 4. Among the top 5 productive institutions, McMaster University (24 publications) was the leading institution, followed by Yale University (11 publications), Harvard University (8 publications). Analysis of centrality via Citaspace indicated that McMaster University in Canada and Harvard University in the US were the 2 most influential institutions in the world. Universities were major research institutions in this field (Fig. 4A).

### 3.4. Analysis of authors

Six hundred twelve authors were included in this study, with high-yield and highly cited authors being distinguished in the current analysis, respectively. In terms of the number of publications, Frey BN (9 publications) from McMaster University in Canada delivered the highest output, followed by Minuzzi L (5 publications), Bertone-Johnson ER (4 publications), Kornstein SG (4 publications), Pilver CE (4 publications), Allega OR (3 publications), Cardoso TD (3 publications), Endicott J (3 publications), Halbreich U (3 publications), and Levy BR (3 publications) (Fig. 3B). Among the authors, Frey BN ranked first in academic influence (i.e., H_index 6, G_index 9, M_index 0.429). Meanwhile, Lieb R from the University of Basel, the oldest university in Switzerland, and Wittchen HU from Tech University Dresden and Ludwig Maximilians University Munchen in Germany were the most cited authors (Table 1).

**Table 1:** Top 10 authors with the most publications, citations, and influential impact in the field of comorbidities in premenstrual disorders.

Rank	Authors	Country/affiliation	Articles	Cited author	Citations	Authors impact	h_index	g_index	m_index
1	Frey BN	Canada/McMaster University	9	Lieb R	57	Frey BN	6	9	0.429
2	Minuzzi L	Brazil/Universidade de São Paulo	5	Wittchen HU	57	Kornstein SG	4	4	0.174
3	Bertone-Johnson ER	U.S./University of Massachusetts Amherst	4	Becker E	39	Pilver CE	4	4	0.308
4	Kornstein SG	U.S./Virginia Commonwealth University	4	Krause P	39	Bertone-Johnson ER	3	4	0.3
5	Pilver CE	U.S./New York University	4	Frey BN	31	Cardoso TD	3	3	0.5
6	Allega OR	U.S./Harvard University	3	Dias RS	24	Endicott J	3	3	0.13
7	Cardoso TD	Brazil/Universidade de São Paulo	3	Lafer B	24	Halbreich U	3	3	0.158
8	Endicott J	U.S./Columbia University	3	Nierenberg AA	24	Levy BR	3	3	0.231
9	Halbreich U	U.S./State University of New York	3	Sachs GS	24	Minuzzi L	3	5	0.273
10	Levy BR	U.S./Yale University	3	Endicott J	22	Soares CN	3	3	0.158

From the publication of Frey BN, the primary focus was on comorbid bipolar disorder and premenstrual dysphoric disorder.^[21–23]^ His recent publication provided an update on the pharmacotherapeutic management of PMDD and proposed promising compounds blocking the synthesis of allopregnanolone treating PMDD.^[24]^ Further, FREY BN generally had the highest academic influence (i.e., H_index 6, G_index 9, M_index 0.429). Lieb R from the University of Basel, the oldest university in Switzerland, and Wittchen HU from Tech University Dresden and Ludwig Maximilians University Munchen in Germany are the most cited authors.

### 3.5. Analysis of productive disciplines and journals

Among the publications, Psychiatry (61.745%), Clinical Neurology (23.49%), Neurosciences (19.463%), Medicine General Internal (10.067%), and Pharmacology Pharmacy (10.067%) were the top 5 disciplines (Table 2). Among these subjects, 100 journals were identified in this research field. Bipolar Disorders (11 publications) ranked first as the most published journal (Table 3). American Journal of Psychiatry is the most frequently cited journal and the tenth most published journal. American Journal of Psychiatry’s Latest Impact Factor is 17.7, which is widely read in psychiatric journals worldwide. Many influential publications have been featured in the journal previously.^[25]^

**Table 2:** Top 10 categories in the field of comorbidities in premenstrual disorders.

Rank	Record	Web of science categories	% of 149
1	Psychiatry	92	61.745
2	Clinical Neurology	35	23.49
3	Neurosciences	29	19.463
4	Medicine General Internal	15	10.067
5	Pharmacology Pharmacy	15	10.067
6	Psychology Clinical	13	8.725
7	Obstetrics Gynecology	12	8.054
8	Psychology	9	6.04
9	Public Environmental Occupational Health	9	6.04
10	Women S Studies	6	4.027

**Table 3:** Top 10 productive Journals and cited journals in the field of comorbidities in premenstrual disorders.

Rank	Journal	Count	% of 149	Cited journal	Frequency	Centrality
1	Bipolar Disorders	11	7.383	American Journal of Psychiatry	88	0.07
2	Archives of Women Mental Health	8	5.369	Journal of Affective Disorders	82	0.03
3	Journal of Clinical Psychiatry	7	4.698	Archives of General Psychiatry	74	0.12
4	European Psychiatry	5	3.356	Journal of Clinical Psychiatry	69	0.01
5	Journal of Affective Disorders	5	3.356	Psychological Medicine	69	0.01
6	Journal of Womens Health	4	2.685	Acta Psychiatrica Scandinavica	60	0.13
7	Cephalalgia	3	2.013	Psychiatry Research	59	0.01
8	Frontiers in Psychiatry	3	2.013	Archives of Womens Mental Health	55	0.01
9	Acta Psychiatrica Scandinavica	2	1.342	Biological Psychiatry	50	0.12
10	American Family Physician	2	1.342	Psychosomatic Medicine	49	0.07

**Figure 5.**
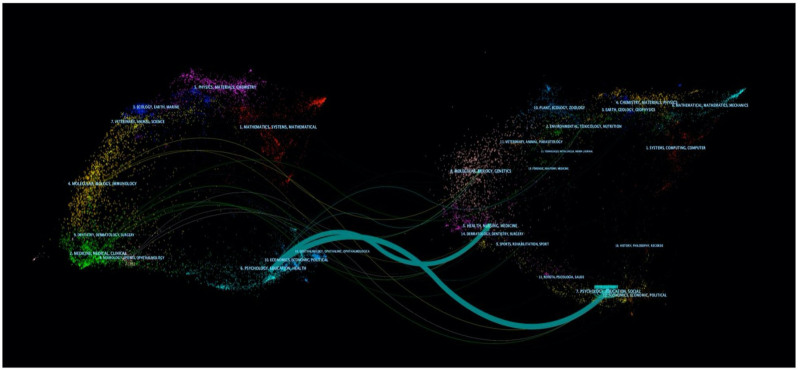
Dual-map of PMDs and comorbidity research. PMDs = premenstrual disorders.

A dual-map overlay analysis was adopted to visualize the citation relationship between journals and reveal the interdisciplinary crossover. Furthermore, the dual-map overlay of journals demonstrated the relationship distribution among journals, citing journals on the left and the right.^[26]^ A green path in Figure 5 gained from Citespace indicated that documents published in health/ nursing/ medicine, dermatology/dentistry/surgery, and psychology/education/social, economics/economic/political journals are often cited by psychology/education/health journals.

### 3.6. Analysis of highly cited papers and co-cited reference

One hundred forty-nine publications were sorted based on their citation frequency, and the top 10 most cited publications were also identified, both were shown in Table 4. The publication timeline of the articles was primarily near the year 2000. Furthermore, the top 10 cited references about frequency and centrality are shown in Table 5. The top 2 papers were “Diagnostic and Statistical Manual of Mental Disorders”^[27]^ and “Diagnostic and Statistical Manual of Mental Disorders: Fifth Edition (DSM-5).”^[28]^ The American Psychiatric Association’s DSM was an authoritative book for the classification of PMDD. Though controversies were raised regarding this diagnostic standard,^[3,29]^ the precise classification of PMDD as a depressive disorder undoubtedly confirmed its type as a mental illness. Besides, “comorbid bipolar and premenstrual dysphoric disorder” was the most researched topic and frequently discussed.^[23]^ BD is a mood disorder that combines depressive episodes with manic or hypomanic episodes. BD exhibited a high comorbidity rate with other psychiatric disorders under a lifetime comorbidity rate of 50% to 70%.^[30]^

**Table 4:** Top 10 articles with the most citations in the field of comorbidities in premenstrual disorders.

Rank	Title	Source title	Publication year	Total citations
1	Prevalence, Incidence and Stability of Premenstrual Dysphoric Disorder in the Community	Psychological Medicine	2002	358
2	Factors Predisposing Women to Chronic Pelvic Pain: Systematic Review	BMJ—British Medical Journal	2006	341
3	Reproductive Hormone Sensitivity and Risk for Depression Across the Female Life Cycle: A Continuum of Vulnerability?	Journal of Psychiatry and Neuroscience	2008	249
4	Sex, Steroids, and Sleep: A Review	Sleep	1999	217
5	Depression in Women	Metabolism-Clinical and Experimental	2005	212
6	The Epidemiology of Perimenstrual Psychological Symptoms	Acta Psychiatrica Scandinavica	2001	195
7	Epidemiology of Depression Throughout the Female Life Cycle	Journal of Clinical Psychiatry	2002	179
8	Anxiety and Depression as Bidirectional Risk Factors for One Another: A Meta-Analysis of Longitudinal Studies	Psychological Bulletin	2017	161
9	Somatic Symptoms and Physiologic Responses in Generalized Anxiety Disorder and Panic Disorder: An Ambulatory Monitor Study	Archives of General Psychiatry	2004	143
10	Plasma Cytokine Profiles in Females With Irritable Bowel Syndrome and Extraintestinal Comorbidity	American Journal of Gastroenterology	2010	128

**Table 5:** Top 10 cited references in the field of comorbidities in premenstrual disorders.

Rank	Cited reference	Year	Count	Centrality
1	Diagnostic and Statistical Manual of Mental Disorders	2011	19	0.17
2	Diagnostic and Statistical Manual of Mental Disorders: Fifth Edition (DSM-5)	2013	12	0.3
3	Increased Illness Burden in Women With Comorbid Bipolar and Premenstrual Dysphoric Disorder: Data From 1099 Women From STEP-BD Study	2017	10	0.01
4	Premenstrual Disorders	2018	7	0
5	Suicidality in Women With Premenstrual Dysphoric Disorder: A Systematic Literature Review	2021	5	0
6	Prevalence and Factors Associated With Premenstrual Dysphoric Disorder: A Community Sample of Young Adult Women	2018	5	0.01
7	Toward the Reliable Diagnosis of DSM-5 Premenstrual Dysphoric Disorder: The Carolina Premenstrual Assessment Scoring System (C-PASS)	2017	5	0
8	Examination of Premenstrual Symptoms as a Risk Factor for Depression in Postpartum Women	2013	5	0.07
9	Premenstrual Dysphoric Disorder Symptoms Following Ovarian Suppression: Triggered by Change in Ovarian Steroid Levels But Not Continuous Stable Levels	2017	4	0
10	Effects of the Menstrual Cycle on Bipolar Disorder	2017	4	0.07

Moreover, BD and PMS patients may experience periodic emotional changes, such as irritability, irritability, anxiety, and low mood. Their ability to control emotions may decrease. Physiological symptoms, such as headaches, chest tightness, and sleep disorders, may occur concurrently. Understanding their common pathogenesis could be an important direction for future research.

### 3.7. Keyword analysis

**Figure 6.**
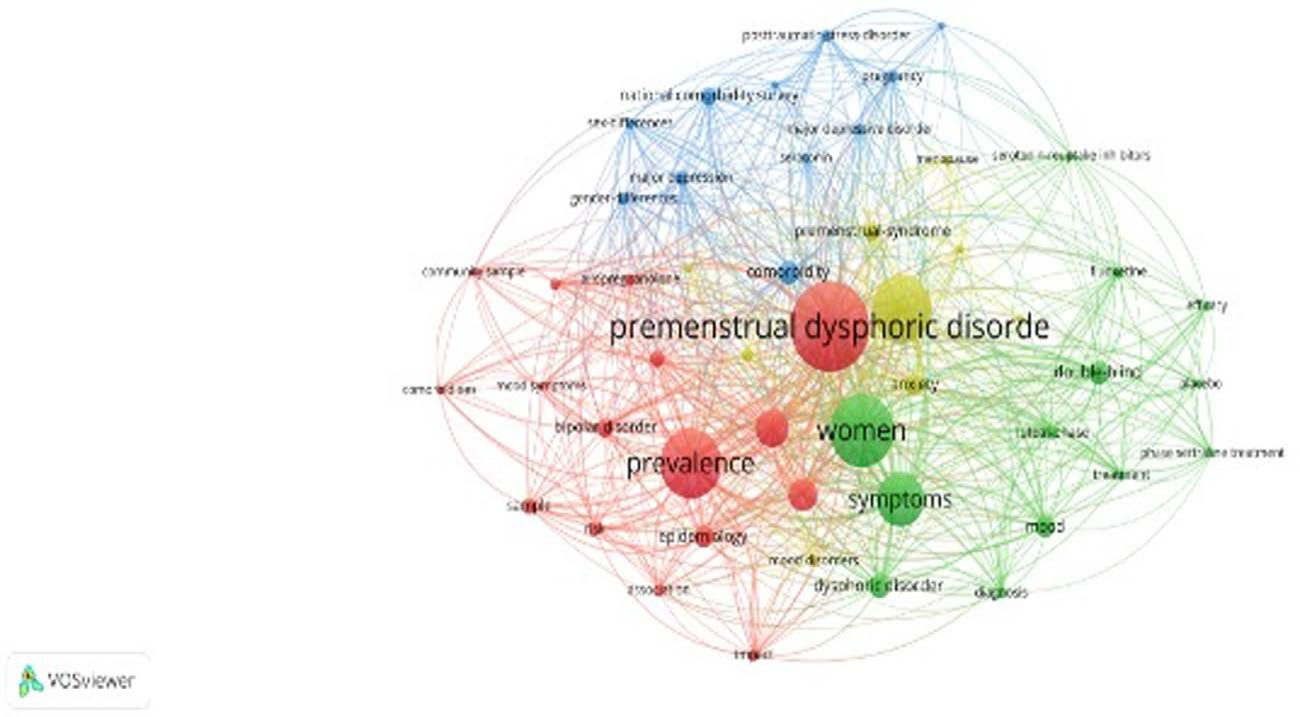
Analysis of research hotspots on PMDs and comorbidity research. PMDs = premenstrual disorders.

**Figure 7.**
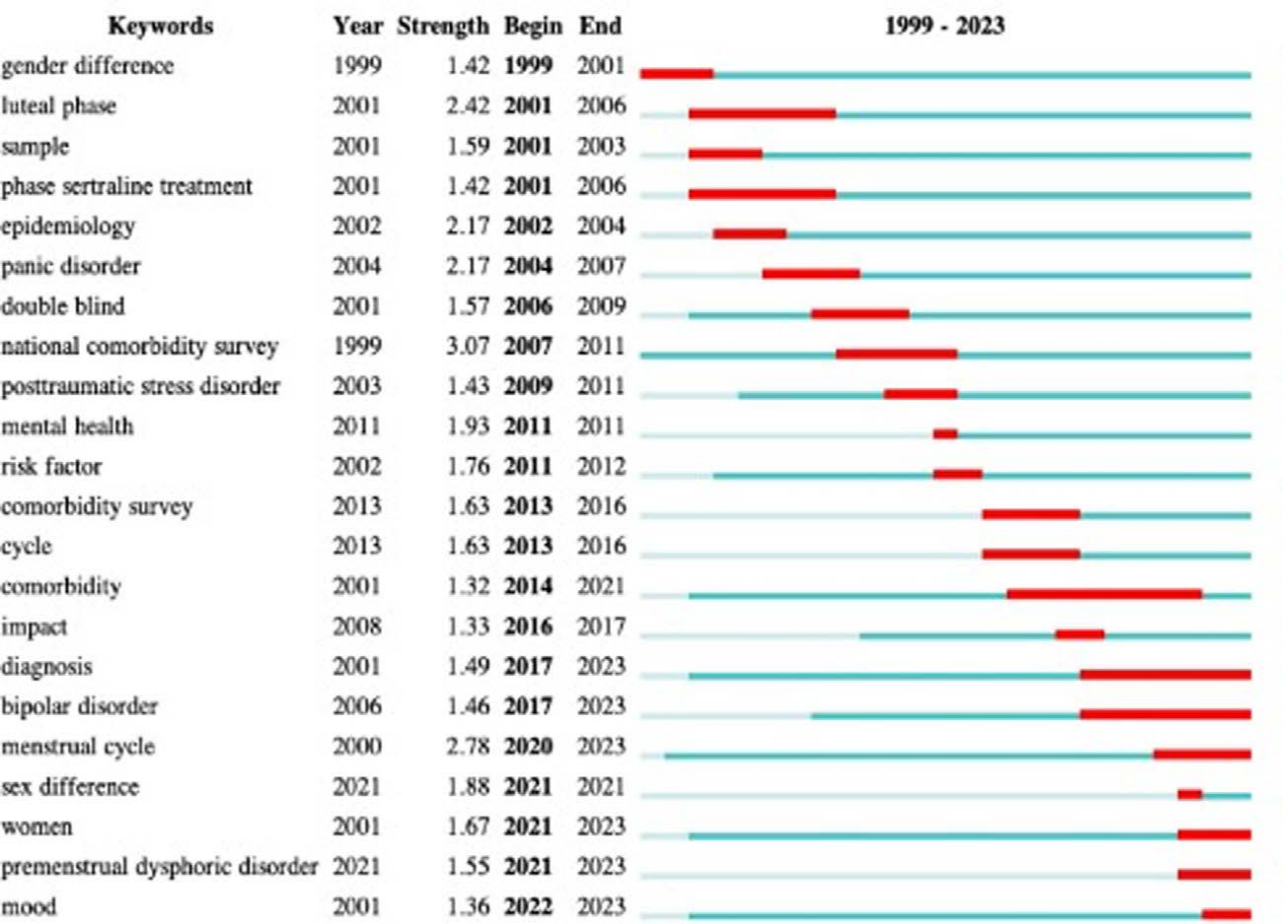
Top 22 keywords with the strongest citation bursts.

The objective of co-occurring keyword analysis served to identify research hotspots. Figure 6 and Table 6 show the top 10 keywords by frequency and centrality. The most frequently occurring keyword is premenstrual dysphoric disorder (n = 49), followed by prevalence (n = 40), women (n = 37), premenstrual syndrome (n = 33), symptom (n = 30), menstrual cycle (n = 29), depression (n = 23), double-blind (n = 15), dysphoric disorder (n = 14), and bipolar disorder (n = 12). Figure 7 presents the top 22 keywords with citation bursts. The blue line indicated the time interval, while the red line indicated the period when a keyword had a burst. The keywords “national comorbidity survey” with the most robust citation bursts appeared in 2007, indicating the importance of the comorbidity survey. The most recent keywords with citation bursts were diagnosis (2017–2023), bipolar disorder (2017–2023), menstrual cycle (2020–2023), women (2021–2023), premenstrual dysphoric disorder (2021–2023), and mood (2022–2023).

**Table 6:** Top 10 keywords in terms of frequency and centrality in the field of comorbidities in premenstrual disorders.

Rank	Frequency	Keywords	Centrality	Keywords
1	49	Premenstrual dysphoric disorder	0.25	Menstrual cycle
2	40	Prevalence	0.25	Double-blind
3	37	Women	0.24	Premenstrual dysphoric disorder
4	33	Premenstrual syndrome	0.22	Premenstrual syndrome
5	30	Symptom	0.21	Depression
6	29	Menstrual cycle	0.15	Prevalence
7	23	Depression	0.15	Gender difference
8	15	Double-blind	0.13	National comorbidity survey
9	14	Dysphoric disorder	0.12	Dysphoric disorder
10	12	Bipolar disorder	0.12	Association

## 4. Discussion

### 4.1. Summary of findings

This study focuses on comorbidities in PMDs research, with 149 publications between 1999 and 2023 were retrieved from the WOSCC database. Over the years, more research effort has been dedicated to the field. Thirty-eight countries, 292 institutions, and 612 authors contributed to PMDs and its comorbidities research. The US was the leading country in this field. Still, McMaster University from Canada delivered the highest publication output, and Frey BN from McMaster University ranked first in academic outputs and influence. Beyond core contributors, citation metrics further highlight the field’s influential work: the American Journal of Psychiatry (impact factor = 17.7, 2023) was the most frequently cited journal (88 citations; Table 3), reflecting its role in disseminating high-quality research on PMD comorbidities. High-cited studies (e.g., Wittchen et al, 2002, 358 citations; Table 4) focused on population-based PMDD prevalence, while recent high-impact work centers on comorbidity mechanisms (e.g., PMDD–BD neurobiology)—indicating a shift from descriptive to translational research.

### 4.2. Research hotspots and emerging areas

**Figure 8.**
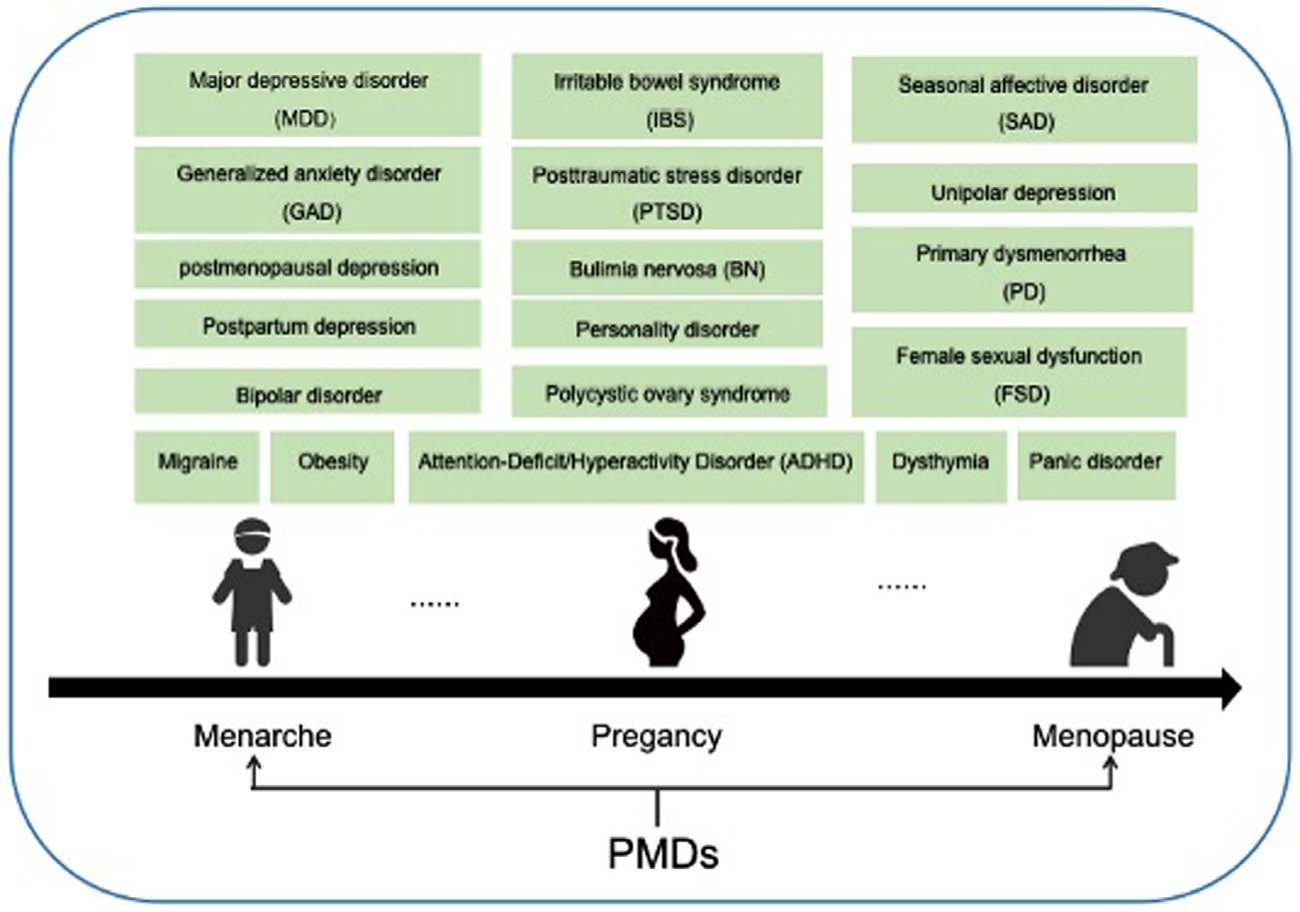
PMDs and comorbidities. PMDs = premenstrual disorders.

PMDs remained inadequately understood, and women with PMDs were more prone to related comorbidity. One hundred forty-nine relevant literatures, as an adequate sample size of global publications, were utilized in the current study to outline and project the trend of this field (Fig. 8).

#### 4.2.1. Temporal evolution of core research hotspots

##### 4.2.1.1. 1999–2010: Foundational prevalence and phenotypic description

This phase focused on establishing the scope of PMD comorbidities through population-based surveys. The keyword “national comorbidity survey” exhibited the strongest citation burst (strength = 3.07, 2007–2011; Fig. 7), reflecting efforts to quantify comorbidity rates at the community level. A landmark 2002 study (Wittchen et al, 358 citations; Table 4) reported the first large-scale data on PMDD prevalence (lifetime stability: 60% over 12 months) and its comorbidity with anxiety (47.4%) and mood disorders (22.9%)—findings that remain foundational for clinical screening.^[31]^ Early work also began linking PMDs to somatic conditions, such as irritable bowel syndrome, though these associations were primarily descriptive.

##### 4.2.1.2. 2011–2016: Diagnostic standardization and comorbidity classification

With the publication of DSM-5 in 2013 (a top-cited reference, 12 citations, centrality = 0.3; Table 5), research shifted to aligning PMD comorbidity criteria with global diagnostic frameworks. The reclassification of PMDD as a distinct “Depressive Disorder” (instead of “not otherwise specified” in DSM-IV) prompted studies to refine comorbidity subtypes—distinguishing psychiatric from somatic comorbidities. Keyword analysis showed rising frequency of “comorbidity” (centrality = 0.12; Table 6) and “association” (centrality = 0.12; Table 6), indicating efforts to map relationships between PMDs and other conditions rather than just documenting their co-occurrence.

##### 4.2.1.3. 2017–2023: Mechanistic exploration and translational research

The most recent phase is defined by a shift from “what co-occurs” to “why it co-occurs,” driven by citation bursts in mechanistically focused keywords: “diagnosis” (2017–2023), “bipolar disorder” (2017–2023), “premenstrual dysphoric disorder” (2021–2023), and “mood” (2022–2023; Fig. 7). High-impact studies in this phase include:

Neurobiological investigations linking comorbid PMDD–BD to disrupted biological rhythms, supported by neuroimaging evidence of shared brain structural abnormalities.^[32]^ BD, and schizophrenia—suggesting shared genetic vulnerabilities. Pharmacotherapeutic advances proposing allopregnanolone synthesis inhibitors as targeted treatments for PMDD, with implications for comorbid mood disorders.^[24]^ The American Journal of Psychiatry (most cited journal, 88 citations; Table 3) played a key role in disseminating this translational work, including debates on PMDD’s diagnostic validity and its mechanistic overlap with BD.^[33]^

#### 4.2.2. PMS/PMDD: core entities and diagnostic nuance

PMS and PMDD remain the primary focus of PMD comorbidity research, with “premenstrual dysphoric disorder” (frequency = 49, Table 6) and “premenstrual syndrome” (frequency = 33, Table 6) as the top 2 keywords. Existing bibliometric analysis^[34,35]^ indicated that PMDD was first discovered in the gynecological population at the beginning of menarche and last seen during menopause. The disorder is also complicated by mental illness, implying significant suffering among patients. A strong correlation between the disorder and the sensitivity of ALLO fluctuations was also unveiled in previous studies.^[36,37]^ In the future, PMDD may be a useful entry point for PMD comorbidity research.

#### 4.2.3. Association, prevalence, and population-specific risks

“Prevalence” (frequency = 40, Table 6) and “women” (frequency = 37, Table 6) are among the most frequent keywords, reflecting the field’s commitment to understanding population-level burden and disparities. The prevalence of PMDD morbidity rates could be up to 73.5%, including a comorbid rate of 47.4%, 22.9%, and 28.4% with anxiety disorder, mood disorder, and somatoform disorder, respectively.^[31]^ Another epidemiology study also pointed out a correlation between the severity of PMS/PMDD and natural disaster-induced PTSD.^[38]^ Based on previous research, several mental and physiological disorders have also been revealed to be associated with PMDD, including bipolar disorder,^[39]^ generalized anxiety disorder,^[40]^ perimenstrual symptoms/syndrome,^[41]^ personality disorders,^[42]^ sexual function, etc. A point to note is that the prevalence of depression in women generally was found to be higher than in men, and comorbidity with PMDs would bring additional harm to patients, which could be difficult to prevent or treat effectively.^[43,44]^ Therefore, it was equally crucial to investigate and sort out the comorbidity relationship and incidence of the disease to develop preventive measures.

#### 4.2.4. Bipolar disorder: the most impactful psychiatric comorbidity

BD has served as a disabling and severe form of psychotic disorder. Clinically, there are 3 subtypes, that is, BD-I, BD-II, and cyclic mood disorder.^[45]^ Epidemiological surveys in China demonstrated that the lifetime prevalence of BD was approximately 0.5%.^[46]^ PMS/PMDD and BD have shown similarities in clinical symptoms and disease course and often co-occurred, severely impacting the social functioning of patients.^[47]^ In clinical practice, both BD patients and PMS patients may experience periodic emotional changes, such as irritability, irritability, anxiety, and low mood. The ability to control emotions was reduced, and a series of physical symptoms such as headache, chest tightness, and sleep disorders may coexist, suggesting a possible pathological and physiological connection between the 2 disorders.^[13]^ In addition, the occurrence of PMS is related to the cyclical fluctuations of sex hormones. The period of rapid changes in estrogen and progesterone, such as adolescence, premenstrual period, postpartum period, and menopause, was also identified as the risky period of BD onset.^[48]^ However, the current research on the common pathogenesis of the 2 may not be adequate and warrants further research in the comorbid conditions.

## 5. Strengths and limitations

To the author’s knowledge, the current study is the first investigation to use bibliometric analysis to study the comorbidities of PMDs. Based on the result, key information, including core authors, countries, and institutions of research in this field, as well as future research trends, were identified. However, due to the limited number of literatures, future research on keyword emergence analysis may be warranted.

## 6. Conclusions

An emerging prevalence of PMS/PMDD, along with its comorbidity, has brought increasing impact on patients. Key information on PMDs comorbidity was thoroughly identified in the current study. The US and Canada were core high-yield countries, and McMaster University and Harvard University were the most influential institutions. Frey BN has made outstanding contributions to the development of this field. In the future, investigation of disease comorbidity epidemiology and psychosomatic diseases may serve in new, meaningful directions.

## Author contributions

**Conceptualization:** Xunshu Cheng.

**Data curation:** Xunshu Cheng, Xiaoying Liu.

**Funding acquisition:** Xiaoying Liu.

**Investigation:** Peijuan Wu.

**Methodology:** Huihao Li, Peijuan Wu.

**Project administration:** Huihao Li.

**Supervision:** Mingzhou Gao, Xingping Ni.

**Visualization:** Xunshu Cheng, Xingping Ni.

**Writing – original draft:** Xunshu Cheng.

**Writing – review & editing:** Mingzhou Gao.
